# Expression of the DNA-dependent protein kinase catalytic subunit is associated with the radiosensitivity of human thyroid cancer cell lines

**DOI:** 10.1093/jrr/rry097

**Published:** 2018-11-23

**Authors:** Makoto Ihara, Kiyoto Ashizawa, Kazuko Shichijo, Takashi Kudo

**Affiliations:** 1 Department of Radioisotope Medicine, Atomic Bomb Disease and Hibakusha Medicine Unit, Atomic Bomb Disease Institute Nagasaki University, 1-12-4 Sakamoto, Nagasaki, Nagasaki, Japan; 2 Department of Tumor and Diagnostic Pathology, Atomic Bomb Disease and Hibakusha Medicine Unit, Atomic Bomb Disease Institute, Nagasaki University, 1-12-4 Sakamoto, Nagasaki, Nagasaki, Japan

**Keywords:** thyroid cancer cell, radiosensitivity, DNA-dependent protein kinase activity, DNA-dependent protein kinase catalytic subunit, predictive assay

## Abstract

The prognosis and treatment of thyroid cancer depends on the type and stage of the disease. Radiosensitivity differs among cancer cells owing to their varying capacity for repair after irradiation. Radioactive iodine can be used to destroy thyroid cancer cells. However, patient prognosis and improvement after irradiation varies. Therefore, predictive measures are important for avoiding unnecessary exposure to radiation. We describe a new method for predicting the effects of radiation in individual cases of thyroid cancer based on the DNA-dependent protein kinase (DNA-PK) activity level in cancer cells. The radiation sensitivity, DNA-PK activity, and cellular levels of DNA-PK complex subunits in five human thyroid cancer cell lines were analyzed *in vitro*. A positive correlation was observed between the D_10_ value (radiation dose that led to 10% survival) of cells and DNA-PK activity. This correlation was not observed after treatment with NU7441, a DNA-PK–specific inhibitor. A significant correlation was also observed between DNA-PK activity and expression levels of the DNA-PK catalytic subunit (DNA-PKcs). Cells expressing low DNA-PKcs levels were radiation-sensitive, and cells expressing high DNA-PKcs levels were radiation-resistant. Our results indicate that radiosensitivity depends on the expression level of DNA-PKcs in thyroid cancer cell lines. Thus, the DNA-PKcs expression level is a potential predictive marker of the success of radiation therapy for thyroid tumors.

## INTRODUCTION

DNA double-stranded breaks (DSBs) are a highly cytotoxic form of DNA damage induced by ionizing radiation [1, 2]. If not repaired, or if repaired incorrectly, DSBs induce mutations, chromosomal aberrations, and cell death. In eukaryotes, DSBs are repaired mainly by homologous recombination (HR) or non-homologous end joining (NHEJ) [3, 4]. In mammalian cells, NHEJ is the major repair pathway. Double-strand DNA–dependent protein kinase (DNA-PK) plays an important role in NHEJ. DNA-PK is a serine/threonine protein kinase, composed of a DNA-dependent protein kinase catalytic subunit (DNA-PKcs) and a Ku70/80 heterodimer. Ku70/80 binds to the ends of DSBs and recruits DNA-PKcs to form an active DNA-PK complex. The active DNA-PK complex is important for cellular radiosensitivity [5], and reduced DNA-PKcs levels, or DNA-PK activity, are associated with increased radiosensitivity [6–8]. We previously reported that mouse DNA-PK was inactivated by heat treatment at 44°C for 15 min [9], because mouse Ku70/80 is heat-sensitive. The radiation sensitivity of heat-treated cells was 2.4-fold that of non-heat–treated cells.

The prognosis and treatment of thyroid cancer depends on the specific cancer type: papillary carcinoma, follicular carcinoma, or anaplastic carcinoma. Eighty percent of thyroid cancers are papillary carcinomas, for which the prognosis is extremely good. Follicular carcinoma is the second most common type of thyroid cancer, accounting for ~17.5% of all thyroid cancers. Approximately 14–39% of all thyroid cancer deaths are due to anaplastic carcinomas, and this form of cancer comprises 2.5% of all thyroid cancers [10, 11]. The prognosis for anaplastic carcinomas is extremely poor. Because thyroid carcinoma cells absorb iodine, radioisotopes such as ^131^I can be used to destroy thyroid cancer cells after surgical removal of the thyroid gland. This approach is used if the cancer is large within the thyroid or if it has spread to lymph nodes or distant areas, such as the lungs and bones [12]. However, prognosis and improvement after irradiation remain controversial [13, 14]. Therefore, predictive measures are important for avoiding unnecessary exposure to radiation.

The sensitivity of thyroid cancer cells to radiation has been reported previously [15, 16]. The DNA damage induced in cells by equal doses of ionizing radiation is the same across different cell types. However, differing capacities for DNA repair following irradiation may result in differences in radiosensitivity among cancer cells. In this report, we describe a new method for predicting the effect of radiation in individual thyroid cancers based on the DNA-PKcs expression levels in the cancer cells.

## MATERIALS AND METHODS

### Cells and irradiation

The human thyroid cells used were papillary carcinoma (TPC-1 and KTC-1), follicular carcinoma (WRO) and anaplastic carcinoma (FRO and KTC-2) cells. Primary culture of thyrocytes was used as a control. Cells were cultured in Dulbecco’s modified Eagle’s medium (high glucose) and Nutrient Mixture F-12 Ham (1:1) supplemented with 5% fetal bovine serum (Equitech-Bio, Inc. Kerrville, TX, USA) in a humidified atmosphere of 5% CO_2_ at 37°C.

Cell survival was measured using the colony-forming assay. In brief, exponentially growing cells were irradiated with 2–10 Gy using a ^137^Cs gamma-ray irradiator (Pony Industry, Chuo-ku, Osaka, Japan) at a dose rate of 0.95 Gy/min at room temperature (20–25°C). Cells were then plated onto 100-mm-diameter culture dishes and incubated at 37°C for 2 weeks. The number of cells per dish was chosen to ensure that ~50 colonies would survive. To inhibit DNA-PK activity, we used 5 μM NU7441 (a specific inhibitor of DNA-PK, AdooQ BioScience LLC, Irvine, CA, USA) [17, 18] and 20 μM wortmannin (an inhibitor of PI-3 kinase) [19]. Cells were pretreated with the inhibitor for 1 h, followed by trypsinization and irradiation. After incubation in a medium containing the inhibitor for 1 day, cells were washed with PBS (–) (137 mM NaCl, 2.7 mM KCl, 10 mM Na_2_HPO_4_ and 1.76 mM KH_2_PO_4_, pH 7.4) and incubated in fresh medium without inhibitor for an additional 2 weeks.

### Assay for DNA-PK activity

DNA-PK activity was measured as described previously [20]. In brief, exponentially growing cells were suspended in low-salt buffer (10 mM HEPES-HCl, 25 mM KCl, 10 mM NaCl, 1.1 mM MgCl_2_ and 0.1 mM EDTA, pH 7.2). After freeze thawing, the cell extract was centrifuged (14 000 × *g* for 5 min at 4°C). The supernatant was collected and used for DNA-PK assays. The reaction mixture contained cell extract (10 μg protein), substrate peptide (an oligopeptide of the human p53 protein: EPPLSQEAFADLLKK), double-stranded DNA, and [γ-^32^P] ATP. After incubation at 30°C for 30 min, the reaction mixture was transferred to a phosphocellulose disk. The disk was washed with 1% acetic acid and distilled water. Radioactivity bound to the disks was measured using a scintillation counter (Hitachi Aloka Medical, Ltd, Mitaka, Tokyo, Japan). DNA-PK kinase activity was determined by evaluating the radioactivity bound to the disks [20].

### Western blot analysis

Protein samples were prepared as described for the DNA-PK activity assay. Protein concentration was determined using a protein assay (Bio-Rad, Hercules, CA, USA) and bovine serum albumin was used as a standard. The same amount of protein was loaded into each well. Protein (10 μg) was separated using SDS-PAGE on a 7.5% gel for Ku70 and Ku80 and a 5% gel for DNA-PKcs and transferred onto nitrocellulose membranes (Hibond ECL; GE Healthcare UK Ltd, Buckinghamshire, England). Proteins were detected with anti-Ku70 (M-19; Santa Cruz Biotechnology, Inc., Santa Cruz, CA, USA), anti-Ku80 (M-20 Santa Cruz Biotechnology), and anti-DNA-PKcs (Ab-2; Neo Markers, Fremont, CA, USA) antibodies (diluted 1:1 000) at 4°C overnight. After washing, the membranes were treated with a secondary antibody conjugated with horseradish peroxidase (Thermo Fisher Scientific, Rockford, IL, USA) (diluted 1:5000) at room temperature (20–25°C) for 3 h and detected using an enhanced chemiluminescent system (ECL; GE Healthcare UK Ltd). The density of the protein bands was measured using densitometry (Photometrics Ltd, Tucson, AZ, USA). The relative expression level was calculated from the density of the protein band. A relative value of 1 indicates the band intensity of the primary thyrocyte cells.

## RESULTS

### Sensitivity of thyroid cancer cells to radiation

**Fig. 1.**
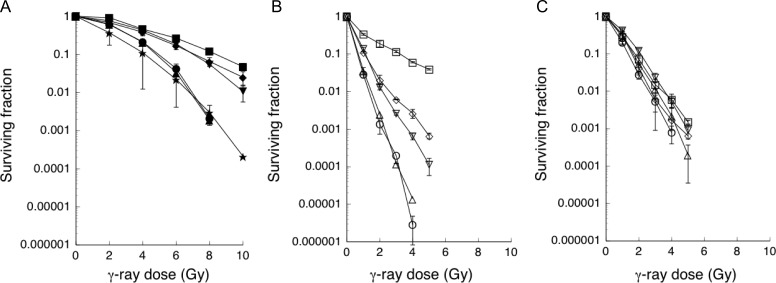
Surviving fraction of non-treated and inhibitor-treated thyroid cancer cells. γ-ray sensitivity was measured using the colony-forming assay. (A) Non-treated control, (B) 5 μM NU7441, (C) 20 μM Wortmannin. The symbols used are: open square, filled square = FRO; open triangle (point down), filled triangle (point down) = WRO; open triangle (point up), filled triangle (point up) = TPC-1; open circle, filled circle = KTC-1; open diamond, filled diamond = KTC-2; filled star = primary thyrocyte. Open symbol indicates inhibitor-treated thyroid cancer cells; filled symbol indicates non-treated thyroid cancer cells. The γ-ray dose resulting in 10% survival (D_10_) was estimated from the survival curves. The data is the average of two or three independent experiments.

We first examined the sensitivity of thyroid cancer cells to radiation using colony-forming assays. The thyroid cancer cells examined clearly separated into three groups. The radiation dose that led to 10% survival (D_10_) was 8.5 Gy for radioresistant cells (FRO), 7.1 Gy for the moderately radiosensitive group (WRO and KTC-2), and 4.85–4.95 Gy for the radiosensitive group (TPC-1 and KTC-1) (Fig. 1A).

### Radiation sensitivity after inhibitor treatment

We then examined the effect of NU7441, a specific inhibitor of DNA-PK, on the sensitivity of cells to radiation. The surviving fraction of NU7441-treated thyroid cancer cells decreased in all cell types (Fig. 1B). From these results, thyroid cancer cells were separated into the three groups of radioresistant, moderately radiosensitive, and radiosensitive groups based on the D_10_ values. The D_10_ was 3.3 Gy for FRO cells (most radioresistant), 1.15 Gy for the moderately radiosensitive group, and 0.65–0.7 Gy for the radiosensitive group (Table 1). Throughout these experiments, the plating efficiency of NU7441-treated cells was lower than that of non-treated cells (Table 1).

**Table 1.** D_10_ and plating efficiency of thyroid cancer cells treated with or without NU7441

	D_10_ (Gy)			Plating efficiency	
	Non-treated	NU7441-treated	Enhancement ratio	Non-treated	NU7441-treated
FRO	8.50	3.30	2.58	0.605	0.052
WRO	7.10	1.15	6.17	0.658	0.477
TPC-1	4.85	0.70	6.93	0.904	0.183
KTC-1	4.95	0.65	7.62	0.702	0.299
KTC-2	7.10	1.15	6.17	0.368	0.173

D_10_ = γ-ray dose at 10% survival, enhancement ratio = D_10_ of non-treated cells/D_10_ of NU7441-treated cells.

Radiation sensitivity after wortmannin treatment was examined. A decrease was noted in the surviving fraction of all wortmannin-treated thyroid cancer cells (Fig. 1C). A similar surviving fraction was observed in all thyroid cancer cell lines examined.

### DNA-PK activity

**Fig. 2.**
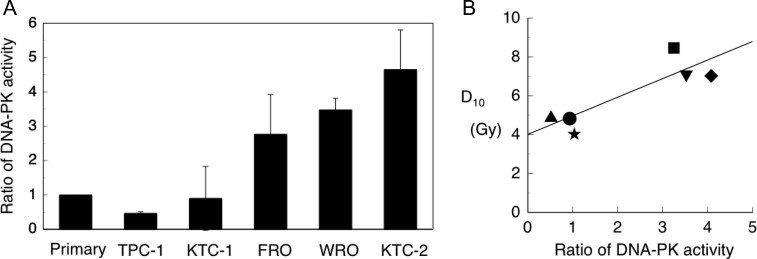
Ratio of DNA-PK activity in thyroid cancer cells. (A) The DNA-PK activity in primary thyrocytes was considered equal to 1. (B) The relationship between D_10_ and relative DNA-PK activity. Symbols used: filled star = primary thyrocyte; filled square = FRO; filled triangle (point down) = WRO; filled triange (point up) = TPC-1; filled circle = KTC-1; and filled diamond = KTC-2.

We next measured DNA-PK activity in the thyroid cancer cell lines. DNA-PK activity was detected using the phosphorylation of serine-15 of the human p53 fragment peptide. We compared the rate of DNA-PK activity in thyroid cancer cell lines with that in primary cultured thyroid cells, which was assigned a value of 1 (Fig. 2A). A moderate correlation was observed between the D_10_ value and DNA-PK activity (*R*^2^ = 0.788) (Fig. 2B).

### Expression of DNA-PK components

**Fig. 3.**
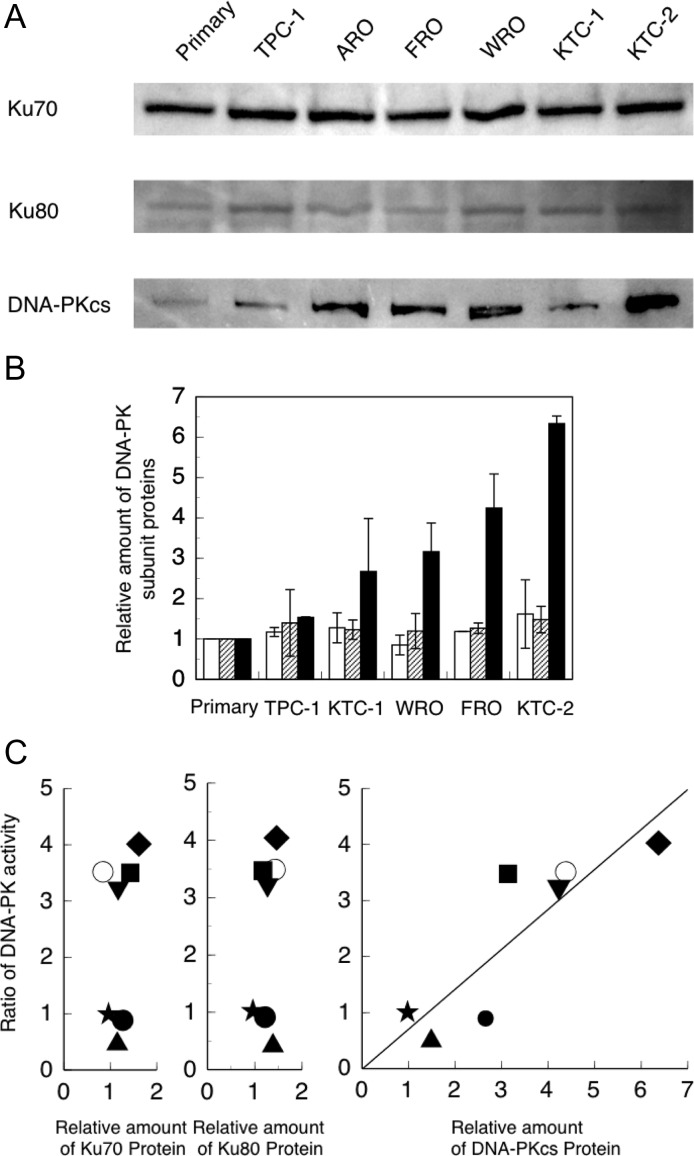
Relationship between DNA-PK activity and DNA-PK subunit expression in non-irradiated cells. (A) Typical results of the Western blot analysis of Ku70, Ku80 and DNA-PKcs. (B) The relative expression levels of Ku70, Ku80 and DNA-PKcs. 
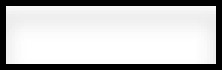
:Ku70 
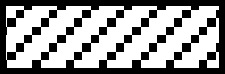
:Ku80 
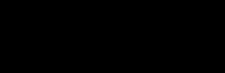
:DNA-PKcs (C) Scatter diagram of the relationship between DNA-PK activity (Fig. 2A) and expression of DNA-PK proteins (Fig. 3B). Symbols used: filled star = primary thyrocyte; filled square = FRO; filled triangle (point down) = WRO; filled triangle (point up) = TPC-1; filled circle = KTC-1; filled diamond = KTC-2; and open circle = ARO. ARO, a colon cancer cell line [20], was included as additional data. The data is the average of two or three independent experiments.

We studied the expression levels of DNA-PK subunit proteins in non-irradiated cell extracts using Western blot analysis. Representative results obtained for Ku70, Ku80 and DNA-PKcs immunoblots are shown in Fig. 3A. Our Western blotting results show the results obtained from ARO cells (Fig. 3A). This cell line was excluded from our analysis because it is of colon cancer, not thyroid cancer, origin [21].

The relative amounts of DNA-PK proteins shown in Fig. 3B are calculated on the basis of Fig. 3A. Ku70 and Ku80 levels were almost unchanged; however, the levels of DNA-PKcs differed between individual thyroid cancer cells. A significant correlation was observed between DNA-PK activity and DNA-PKcs expression (*y* = 0.6903446*x*, *R*^2^ = 0.921295). Including the data for ARO, a colon cancer cell line [21] that responded similarly to the radioresistant group, did not change the relationship observed between DNA-PKcs expression and radiosensitivity (*y* = 0.7106501*x*, *R*^2^ = 0.9362435) (Fig. 3C).

## DISCUSSION

Consistent with previous reports, the radiosensitivity of the thyroid cancer cell lines examined varied (Fig. 1A) [15, 16]. Using the D_10_ value of the cells, we separated the thyroid cancer cells into three groups: the radiation-sensitive group (TPC-1, KTC-1), the radiation-resistant group (WRO, KTC-2), and the extremely radiation-resistant group (FRO). Previously, Namba *et al.* reported that FRO cells are resistant to radiation [15]. Treatment with NU7441 increased radiation sensitivity in all thyroid cancer cell lines examined (Fig. 1B, Table 1). The enhancement ratio (D_10_ of non-treated cells/D_10_ of NU7441-treated cells) of TPC-1, KTC-1, WRO and KTC-2 was >6. This indicates that NU7441 treatment induces 6-fold greater radiation sensitivity in these cells. This suggests that the major DSB repair mechanism in these cells is the NU7441-sensitive DNA-PK–mediated repair mechanism (NHEJ). Furthermore, these results indicate that, with the exception of FRO, the relative contributions of HR (NU7441-resistant repair) and NHEJ (NU7441-sensitive repair) to DSB repair are identical in the thyroid cancer cell lines examined. The TPC-1 and KTC-1 cell lines are derived from papillary carcinoma and belong to the radiation-sensitive group. The WRO and KTC-2 cell lines are derived from follicular and anaplastic carcinoma, respectively, and belong to the radiation-resistant group. These results indicate that radiosensitivity and the contribution of NHEJ in thyroid cancer cells do not depend on the pathological cancer type. The enhancement ratio of FRO was 2.58, suggesting that the contribution of HR in DSB repair is relatively higher in FRO cells than in the other thyroid cancer cell lines examined.

To investigate the relationship between DNA-PK activity and radiosensitivity in five thyroid cancer cell lines, we compared the D_10_ values and DNA-PK activity. We observed a good correlation between D_10_ values and DNA-PK activity (Fig. 2B). We also studied the effect of wortmannin, a specific PI-3 kinase inhibitor [19], on the γ-ray sensitivity of thyroid cancer cells. In these experiments, the radiosensitivity of the examined thyroid cancer cell lines was relatively unchanged following wortmannin treatment (Fig. 1C). In accordance with previous reports [22, 23], these results indicate that among the different thyroid cancer cells examined, the basic repair mechanisms, NHEJ and HR, are identical and inhibited by wortmannin.

We also compared DNA-PK activity and the expression levels of individual DNA-PK subunit proteins. Ku70 and Ku80 levels were similar across all the various thyroid cancer cell lines (Fig. 3B). Additionally, no correlation was observed between DNA-PK activity and the expression of Ku70 and Ku80 proteins (Fig. 3C). However, a significant difference was observed in DNA-PKcs expression in the extracts from the various cell lines (Fig. 3B). Furthermore, a correlation was observed between DNA-PK activity and the expression of DNA-PKcs (*y* = 0.6903446*x*, *R*^2^ = 0.921295) (Fig. 3C), indicating that the DNA-PK activity depends on the expression level of DNA-PKcs. Taken together, these results show that the radiosensitivity of thyroid cancer cells *in vitro* depends on the level of DNA-PKcs expression.

Including the data for ARO, a colon cancer cell line [21] that responded similarly to the radiosensitive group, did not change the relationship observed between DNA-PKcs expression and radiosensitivity (*y* = 0.7106501*x*, *R*^2^ = 0.9362435) (Fig. 3C). This suggests that the relationship between DNA-PKcs and radiation sensitivity is common among cancer cells.

**Fig. 4.**
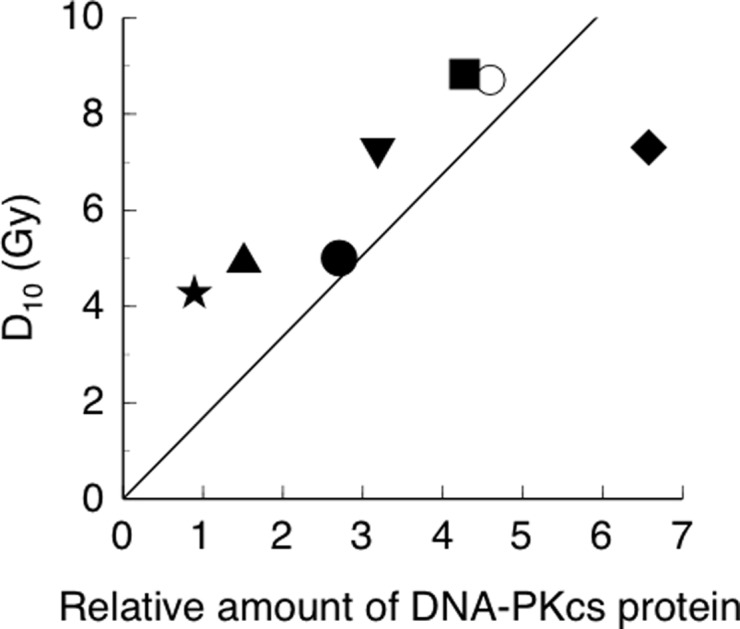
Relationship between expression of DNA-PKcs and D_10._ Symbols used: filled star = primary thyrocyte; filled square = FRO; filled triangle (point down) = WRO; filled triange (point up) = TPC-1; filled circle = KTC-1; filled diamond = KTC-2; and open circle = ARO.

To confirm our results, we determined the relationship between DNA-PKcs expression and the D_10_ value. As indicated in Fig. 4, a good correlation was observed (*y* = 1.689396*x*, *R*^2^ = 0.9015683E-1), indicating that the expression of DNA-PKcs correlates with not only DNA-PK activity but also with the radiation sensitivity of cells.

The relationship between DNA-PK activity and the expression of DNA-PK complex proteins has been studied previously [24–34]. Someya *et al.* found that DNA-PK activity is associated with chromosomal instability, risk of cancer [24], distant metastasis, and poor prognosis [25]. Shintani *et al.* reported that expression of DNA-PKcs after radiation treatment correlates to radiation resistance (D_10_) in oral squamous cell carcinoma [26]. Noguchi *et al.* reported that high expression of DNA-PKcs correlates with a chemoradiotherapy effect in esophageal cancer [27]. DNA-PKcs could be a predictive marker of recurrence after radiotherapy in prostate cancer [28, 29], and DNA-PKcs expression may have prognostic and predictive significance in epithelial ovarian cancer [30]. According to Hsu *et al.*, clinical studies have indicated that expression and activity of DNA-PKcs is correlated with cancer progression and response to treatment [31]. Therefore, DNA-PK expression levels correlate with radiation sensitivity. However, Zhao *et al.* described a significant correlation between DNA-PK activity and Ku70 expression in esophageal cancer cell lines [32]. This suggests that the measurement of DNA-PK activity and/or Ku70 expression may provide a useful way to predict radiation sensitivity. However, no significant association was observed between DNA-PKcs expression levels and radiosensitivity [33, 34]. This apparent lack of association may be because of the use of different tissue types and methods in the various studies.

NU7441-treated FRO cells are extremely resistant to γ-ray irradiation. This suggests that NU7441 does not inhibit repair mechanisms other than NHEJ in FRO cells. The survival curve of NU7441-treated FRO cells is biphasic, similar to that of Ku-deficient cells [35], and the slow component of biphasic repair is due to HR. These results suggest the preferential use of HR for the repair of DSBs in FRO cells. This may explain the resistance to γ-ray irradiation following NU7441 treatment. The plating efficiency of NU7441-treated thyroid cancer cells was markedly reduced compared with that of untreated cells (Table 1). The plating efficiency of NU7441-treated FRO cells, in particular, was extremely low (0.052) compared with that of WRO (0.477) and KTC-1 (0.298). These results suggest that NU7441 exerts its effects through additional mechanisms, such as toxicity.

TPC-1 and KTC-1, of papillary cancer origin, are radiation-sensitive. DNA-PK activity and the expression levels of DNA-PKcs in these cells are low. The reason for low DNA-PKcs levels in these cells remains to be fully elucidated. Ahmed *et al.* reported that the cells of cultured skin fibroblasts from patients with papillary (differentiated) thyroid carcinomas exhibit enhanced radiosensitivity [36]. It is unknown whether all papillary carcinomas are radiation-sensitive. However, the development of method(s) for identifying low DNA-PKcs expression can avoid unnecessary exposure of patients to radiation.

We suggest that DNA-PKcs can be a potential biomarker for predicting radiosensitivity both *in vitro* and *in vivo*. The limitation of this study is that the results presented come from an established cell line. Solid tumors, including thyroid cancer, often have tumor microenvironments [37]; therefore, the intra-tumoral circumstance is not homogeneous. In cancer cells, genetic mutations and metabolic alterations occur because of the surrounding conditions such as energy availability and the stroma environment. Thus, the correlation may change *in vivo* if DNA-PK activity is affected by tumor microenvironments. Clinically, DNA repair activity varies in both spatial and temporal terms, and the expression of DNA-PKcs shows intra-tumor heterogeneity even in the same tumor, as described by Tonotsuka *et al.* [38]. Further studies are needed using clinical samples.

In conclusion, the sensitivity of thyroid cancer cells to radiation correlates with their DNA-PKcs expression levels *in vitro*. Cells that express lower DNA-PKcs levels, such as TPC-1 and KTC-1, are radiation-sensitive, and cells that express higher levels of DNA-PKcs, such as FRO and KTC-2, are radiation-resistant. However, our present results were obtained from a limited number of laboratory strains of cultured thyroid cancer cells. Therefore, additional studies, such as those exploring the relationships between the DNA-PKcs expression level and radiation sensitivity using clinical samples, are required. Nonetheless, our results show that the expression level of DNA-PKcs is a potential marker for predicting the radiosensitivity of thyroid cancer cells.

## References

[1] van Gent D-C, Hoeijmakers J-H, Kanaar R Chromosomal stability and the DNA double-stranded break connection. Nat Rev Genet 2001;2:196–206. 11256071 10.1038/35056049

[2] Dikomey E, Borgmann K, Brammer I et al Molecular mechanisms of individual radiosensitivity studied in normal diploid human fibroblasts. Toxicology 2003;193:125–35. 14599772 10.1016/s0300-483x(03)00293-2

[3] Khanna K-K, Jackson S-P DNA double-strand breaks: signaling, repair and the cancer connection. Nat Genet 2001;27:247–54. 11242102 10.1038/85798

[4] Burma S, Chen D-J Role of DNA–PK in the cellular response to DNA double-strand breaks. DNA Repair 2004;3:909–18. 15279776 10.1016/j.dnarep.2004.03.021

[5] Mahaney B-L, Meek K, Lees-Miller S-P Repair of ionizing radiation–induced DNA double-strand breaks by non-homologous end-joining. Biochem J 2009;417:639–50. 19133841 10.1042/BJ20080413 PMC2975036

[6] Nicolas N, Finnie N-J, Cavazzana-Calvo M et al Lack of detectable defect in DNA double-strand break repair and DNA-dependent protein kinase activity in radiosensitive human severe combined immunodeficiency fibroblasts. Eur J Immunol 1996;26:1118–22. 8647176 10.1002/eji.1830260524

[7] Peng Y, Zhang Q, Nagasawa H et al Silencing expression of the catalytic subunit of DNA-dependent protein kinase by small interfering RNA sensitizes human cells for radiation-induced chromosome damage, cell killing, and mutation. Cancer Res 2002;62:6400–4. 12438223

[8] Daido S, Yamamoto A, Fujiwara K et al Inhibition of the DNA-dependent protein kinase catalytic subunit radiosensitizes malignant glioma cells by inducing autophagy. Cancer Res 2005;65:4368–75. 15899829 10.1158/0008-5472.CAN-04-4202

[9] Ihara M, Takeshita S, Okaichi K et al Heat exposure enhances radiosensitivity by depressing DNA-PK kinase activity during double strand break repair. Int J Hyperthermia 2014;30:102–9. 24571173 10.3109/02656736.2014.887793

[10] Kebebew E, Greenspan FS, Clark OH et al Anaplastic thyroid carcinoma treatment outcome and prognostic factors. Cancer 2005;103:1330–5. 15739211 10.1002/cncr.20936

[11] Nguyen Q-T, Lee E-J, Huang M-G et al Diagnosis and treatment of patients with thyroid cancer. Am Health Drug Benefits 2015;8:30–40. PMC4415174 25964831

[12] Ain K-B Anaplastic thyroid carcinoma: behavior, biology, and therapeutic approaches. Thyroid 1998;8:715–26. 9737368 10.1089/thy.1998.8.715

[13] Lee N, Tuttle M The role of external beam radiotherapy in the treatment of papillary thyroid cancer. Endocr Relat Cancer 2006;13:971–7. 17158749 10.1677/ERC-06-0039

[14] Wilson P-C, Millar B-M, Brierley J-D The management of advanced thyroid cancer. Clin Oncol 2004;16:561–8. 10.1016/j.clon.2004.08.009 15630850

[15] Namba H, Hara T, Tukazaki T et al Radiation-induced G1 arrest is selectively mediated by the p53-WAF1/Cip1 pathway in human thyroid cells. Cancer Res 1995;55:2075–80. 7743505

[16] Miller R-C, Hiraoka T, Kopecky K-J et al Sensitivity to radiation of human normal, hyperthyroid, and neoplastic thyroid epithelial cells in primary culture. Radiat Res 1987;111:81–91. 3602357

[17] Tavecchio M, Munck J-M, Cano C et al Further characterisation of the cellular activity of the DNA-PK inhibitor, NU7441, reveals potential cross-talk with homologous recombination. Cancer Chemother Pharmacol 2012;69:155–64. 21630086 10.1007/s00280-011-1662-4

[18] Ciszewski WM, Tavecchio M, Dastych J et al DNA-PK inhibition by NU7441 sensitizes breast cancer cells to ionizing radiation and doxorubicin. Breast Cancer Res Treat 2014;143:47–55. 24292814 10.1007/s10549-013-2785-6

[19] Chernikova S-B, Wells R-L, Elkind M-M Wortmannin sensitizes mammalian cells to radiation by inhibiting the DNA-dependent protein kinase-mediated rejoining of double-strand breaks. Radiat Res 1999;151:159–66. 9952300

[20] Ihara M, Suwa A, Komatsu K et al Heat sensitivity of double-stranded DNA-dependent protein kinase (DNA-PK) activity. Int J Radiat Biol 1999;75:253–8. 10072187 10.1080/095530099140717

[21] Schweppe R-E, Klopper J-P, Korch C et al Deoxyribonucleic acid profiling analysis of 40 human thyroid cancer cell lines reveals cross-contamination resulting in cell line redundancy and misidentification. J Clin Endocrinol Metab 2008;93:4331–41. 18713817 10.1210/jc.2008-1102 PMC2582569

[22] Allen C, Halbrook J, Nickoloff J-A Interactive competition between homologous recombination and non-homologous end joining. Mol Cancer Res 2003;1:913–20. 14573792

[23] Sak A, Stueben G, Groneberg M et al Targeting of Rad51-dependent homologous recombination: implications for the radiation sensitivity of human lung cancer cell lines. Br J Cancer 2005;92:1089–97. 15785736 10.1038/sj.bjc.6602457 PMC2361929

[24] Someya M, Sakata K, Matsumoto Y et al The association of DNA-dependent protein kinase activity with chromosomal instability and risk of cancer. Carcinogenesis 2006;27:117–22. 16000400 10.1093/carcin/bgi175

[25] Someya M, Sakata K, Matsumoto Y et al The association of DNA-dependent protein kinase activity of peripheral blood lymphocytes with prognosis of cancer. Br J Cancer 2011;104:1724–9. 21559021 10.1038/bjc.2011.158 PMC3111168

[26] Shintani S, Mihara M, Li C et al Up-regulation of DNA-dependent protein kinase correlates with radiation resistance in oral squamous cell carcinoma. Cancer Sci 2003;94:894–900. 14556663 10.1111/j.1349-7006.2003.tb01372.x PMC11160163

[27] Noguchi T, Shibata T, Fumoto S et al DNA-PKcs expression in esophageal cancer as a predictor for chemoradiation therapeutic sensitivity. Ann Surg Oncol 2002;9:1017–122. 12464596 10.1007/BF02574522

[28] Bouchaert P, Guerif S, Debiais C et al DNA-PKcs expression predicts response to radiotherapy in prostate cancer. Int J Radiat Oncol Biol Phys 2012;84:1179–85. 22494583 10.1016/j.ijrobp.2012.02.014

[29] Molina S, Guerif S, Garcia A et al DNA-PKcs expression is a predictor of biochemical recurrence after permanent iodine 125 interstitial brachytherapy for prostate cancer. Int J Radiat Oncol Biol Phys 2016;95:965–72. 27113564 10.1016/j.ijrobp.2016.02.015

[30] Abdel-Fatah T-M-A, Arora A, Moseley P et al ATM, ATR and DNA-PKcs expressions correlate to adverse clinical outcomes in epithelial ovarian cancers. BBA Clin 2014;2:10–7. 26674120 10.1016/j.bbacli.2014.08.001 PMC4633921

[31] Hsu F-M, Zhang S, Chen B-P-C Role of DNA-dependent protein kinase catalytic subunit in cancer development and treatment. Transl Cancer Res 2012;1:22–34. 22943041 10.3978/j.issn.2218-676X.2012.04.01 PMC3431019

[32] Zhao H-J, Hosoi Y, Miyachi H et al DNA-dependent protein kinase activity correlates with Ku70 expression and radiation sensitivity in esophageal cancer cell lines. Clin Cancer Res 2000;6:1073–8. 10741736

[33] Bjork-Eriksson T, West C, Nilsson A et al The immunohistochemical expression of DNA-PKcs and Ku (p70/p80) in head and neck cancers: relationships with radiosensitivity. Int J Radiat Oncol Biol Phys 1999;45:1005–10. 10571209 10.1016/s0360-3016(99)00268-0

[34] Lee S-W, Cho K-J, Park J-H et al Expressions of Ku70 and DNA-PKcs as prognostic indicators of local control in nasopharyngeal carcinoma. Int J Radiat Oncol Biol Phys 2005;62:1451–7. 16029807 10.1016/j.ijrobp.2004.12.049

[35] Fukushima T, Takata M, Morrison C et al Genetic analysis of the DNA-dependent protein kinase reveals an inhibitory role of Ku in late S–G2 phase DNA double-strand break repair. J Biol Chem 2001;276:44413–18. 11577093 10.1074/jbc.M106295200

[36] Ahmed M, AI-Khodary F, Khan BA et al Cellular radiosensitivity of patients with papillary thyroid cancer. Radiother Oncol 1999;53:85–8. 10624858 10.1016/s0167-8140(99)00136-x

[37] Ciavardelli D, Bellomo M, Consalvo A et al Metabolic alterations of thyroid cancer as potential therapeutic targets. Biomed Res Int 2017;2017:2545031 10.1155/2017/2545031. 29234677 PMC5694990

[38] Tonotsuka N, Hosoi Y, Miyazaki S et al Heterogeneous expression of DNA-dependent protein kinase in esophageal cancer and normal epithelium. Int J Mol Med 2006; 18: 441–7. 16865228

